# Family Cluster of Mayaro Fever, Venezuela

**DOI:** 10.3201/eid1007.030860

**Published:** 2004-07

**Authors:** Jaime R. Torres, Kevin L. Russell, Clovis Vasquez, Robert B. Tesh, Rosalba Salas, Douglas M. Watts

**Affiliations:** * Universidad Central de Venezuela, Caracas, Venezuela; † U.S. Naval Health Research Center, San Diego, California, USA; ‡ Ministry of Health and Social Welfare, Caracas, Venezuela; § University of Texas Medical Branch at Galveston, Galveston, Texas, USA; ¶ Caribbean Epidemiology Centre, Republic of Trinidad and Tobago

**Keywords:** Mayaro, Arbovirus, ELISA, enzyme-linked immunosorbent assay, Venezuela, Febrile Illness, Tropical Illnesses, dispatch

## Abstract

A cluster of protracted migratory polyarthritis involving four adult family members occurred in January 2000 after a brief overnight outing in a rural area of Venezuela. Laboratory testing demonstrated Mayaro virus as the cause of the cluster. These results documented the first human cases of Mayaro virus in Venezuela.

Mayaro virus (MAYV), the cause of Mayaro fever, is a member of the genus *Alphavirus*, family *Togaviridae*, and is closely related to Chikungunya, O’nyong-nyong, Ross River, Barmah Forest, and Sindbis viruses (*1*–*3*). Infection by these viruses produce similar clinical illnesses in humans (*4*–*8*). Mayaro fever is typically a denguelike acute febrile illness 3–5 days in duration, characterized by headache, retroorbital pain, arthralgias, arthritis, myalgias, vomiting, diarrhea, and rash (*8*). However, joint involvement in Mayaro fever may persist for several months and in some cases precede the fever. Moderate-to-severe polyarthritis, occasionally incapacitating, is a prominent feature of the disease (*8*).

MAYV is enzootic in South America, where the suspected vectors are forest-dwelling *Haemagogus* mosquitoes, and the vertebrate hosts are marmosets and other nonhuman primates (*8*). Most human cases occur sporadically and involve persons who work or reside in humid tropical forests (*8*,*9*). Nevertheless, several small outbreaks of Mayaro fever have been described in residents of rural communities of the Amazon region of Brazil, Bolivia, and Peru (*8*–*10*). Airborne transmission has been reported among laboratory personnel (*11*). Although MAYV is enzootic in several South American countries, this report describes the first human cases of Mayaro fever in Venezuela. The cases occurred among members of the same family after a single day’s exposure to a semirural forested area. The observations we report were made in response to the Ministry of Health’s request to determine the cause of the cluster of cases.

## The Study

Clinical cases resembling dengue fever were studied in the vicinity of Padrón Agriculture Station, in Miranda State, north-central Venezuela (10°13′22″ N; 66°17′56″ W; 50 m elevation), a location where entomologic and epidemiologic studies on Venezuelan equine encephalitis virus (VEEV) and other arboviruses were conducted from 1997 to 1998 (*12*). This area, originally covered by lowland tropical rain forest, was converted into cacao (*Theobroma cacao*) plantations. Indigenous tall trees (*Erythrina poeppigiana*, *Ceiba pentandra*, *Ficus* sp., *Hura crepitans*, *Bauhinia* sp.), were preserved so that the area resembled a natural forest habitat. The mean temperature and annual rainfall were 27.2°C and 2,324 mm, respectively, with the rainy season normally lasting from May to December.

Four adult members of the same family (age range 26–58 years), spent a single night together in early January 2000 near the Padrón Agriculture Station. While sharing an outdoor dinner, they were frequently bitten by mosquitoes. Three days later, all four had a sudden onset of malaise, fever (up to 40°C), retroocular pain, generalized headache, conjunctival suffusion, flushing of the face and neck, myalgias, and severe incapacitating polyarthralgias and polyarthritis which mainly involved the small joints of the hands, wrists, ankles, and toes. Joints became swollen and tender, but effusion was not evident. Pain was intense and worsened with motion. Limbs felt weak and very sensitive to touch. Joint stiffness in the morning and after inactivity was a prominent complaint. On day 5 of illness, a rapidly spreading maculopapular rash developed, which involved neck, trunk, and limbs. The rash persisted for 2 days, followed by desquamation. In three of the patients, painful cervical, preauricular, and retroauricular lymphadenopathies occurred and lasted approximately 2 weeks. Beyond week 2 of illness, only severe joint symptoms and lower limb hyperesthesias persisted, but they steadily resolved during a 6-month period. Clinical laboratory results were unremarkable except for a transient and mild increase in erythrocyte sedimentation rate and serum levels of alanine aminotransferase, and a moderate lymphocytosis.

Serum samples were obtained 3 months after onset of symptoms, when the patients were first seen at consultation by one of the authors. Samples were also collected an additional 3 months after the initial samples were collected. The patients’ initial signs and symptoms resembled a classical febrile syndrome, and the patients had a history of suspected risk for arboviral infection. Therefore, all samples were tested initially at a 1:100 dilution for immunoglobulin (Ig) M antibodies to MAYV; VEEV; dengue viruses (DENV) 1, 2, 3, and 4; yellow fever virus (YFV); and Oropouche virus (OROV) by using an IgM antibody-capture enzyme-linked immunosorbent assay (MACEIA) (*9*,*12*). Reactive samples were subsequently retested for IgM antibody at serial dilutions ranging from 1:200 through 1:102,400 to determine end-point titers. Serum samples were also tested by an indirect ELISA for IgG antibodies to the above-mentioned viruses (*9*,*13*). A patient with MAYV disease was defined as a person with compatible clinical illness, for whom IgM antibody titers to MAYV and VEEV were >400 and <100, respectively.

## Results

Serologic results indicated that three of the four family members had a MAYV viral infection. Assay of serum samples obtained 3 months after onset of symptoms from the three members showed high specific Mayaro viral IgM antibody ranging from 3,200 to 6,400 and IgG antibody titers ranging from 6,400 to 12,800 (Table). Testing of samples from the fourth patient were positive for MAYV IgG antibody only. Subsequent samples taken approximately 3 months later were IgM negative but remained positive for MAYV IgG antibody. All patients were negative for VEEV IgM antibody but had VEEV IgG antibody ranging from 100 to 800. Assay results for DENV and OROV IgM and IgG were negative. Simlarly, the patients were negative for YFV IgM antibody but had IgG antibody to this virus.

**Table:** Mayaro and Venezuelan equine encephalitis viral IgM and IgG antibodies demonstrated by an antibody-capture ELISA in serum samples obtained from three Venezuelan family members^a^

Patient	Antibody titers^b^								
	Convalescent-phase^c^				Late convalescent–phase^d^				
	MAYV		VEEV		MAYV		VEEV		
	IgM	IgG	IgM	IgG	IgM	IgG	IgM	IgG	
1	3,200	12,800	0	400	0	12,800	0	400	
2	6,400	12,800	0	800	0	12,800	0	800	
3	6,400	6,400	0	400	0	6,400	0	100	

^a^MAYV, Mayaro virus; VEEV, Venezuelan equine encephalitis virus; IgM, IgG, immunoglobulin M, G; ELISA, enzyme-linked immunosorbent assay. ^b^Reciprocal of highest serum dilution at which a positive result occurred. ^c^Three months after onset of illness. ^d^Six months after onset of illness.

## Conclusions

MAYV has not been isolated in Venezuela, but isolates have been obtained from humans, wild vertebrates, and mosquitoes in Colombia, Brazil, Suriname, Guyana, French Guiana, Peru, United States, and Bolivia (*2*,*8*–*10*,*14*–*17*). In addition, serologic survey data suggest that MAYV infection is relatively common among humans in rural populations of northern South America and the Amazon River basin (*2*,*8*,*9*,*14*–*16*). This virus is believed to be maintained in a sylvan cycle involving wild vertebrates, such as nonhuman primates and possibly birds, and *Haemagogus* mosquitoes (*2*,*8*,*18*). Three species of that genus, *H. celeste*, *H. equinus*, and *H.lucifer*, have recently been identified in the area where the Venezuela patients acquired MAYV infection (*12*). Also, the red howler monkey (*Alouatta seniculus*), a suspected host of MAYV in nature, is common in the area. Thus, the results of this study suggest that these first documented cases of Mayaro fever in Venezuela were acquired during an overnight outing in a rural area where MAYV may have been circulating in a cycle involving *Haemagogus* mosquitoes and red howler monkeys.

Of note, convalescent-phase serum samples from an additional unrelated patient (a 40-year-old woman who lived in a nearby rural location), obtained approximately 4 months after she had recovered from a self-limited febrile illness with polyarthritis similar to that described in the patients involved in this report, showed high (25,600) MAYV IgG antibody titers. These samples were negative for IgM antibody, however, which provides further evidence that MAYV was enzootic in the area.

As observed in this study, Mayaro fever cases are usually sporadic and occur in persons with a history of recent activities in humid tropical forests (*4*,*8*,*9*,*19*). Typically, Mayaro fever ensues approximately 1 week after infection (*4*,*8*). However, shorter incubation periods, similar to those observed in these Venezuelan cases, are occasionally observed. Members of the family described in this outbreak had symptoms and clinical courses consistent with previously documented MAYV patients. Abrupt onset of fever, frontal headaches, myalgias, and incapacitating arthralgias were predominant complaints. A maculopapular rash, also a common manifestation in up to 90% of children and 50% of adults (*4*,*8*,*9*,*18*), was prominent in these patients, lasting 2 days and followed by desquamation. Up to one third of patients initially have nausea, vomiting, and diarrhea (*4*,*8*,*9*,*18*,*19*), but these symptoms were not experienced in this family.

Little information is available on the kinetics of MAYV IgM antibodies for Mayaro fever patients during long-term follow-up examinations. While obtaining acute-phase blood samples from the patients in this study was bit possible, existing data indicate that detectable IgM antibody develops after viremia subsides, which is usually 4–5 days after the onset of symptoms (*9*,*19*). Our data indicated that IgM antibody persisted for >3 but <6 months for our patients. These are the first documented data on the persistence of IgM antibody following a Mayaro viral infection and will be useful for interpreting diagnostic test results. To our knowledge, this is the first report of human cases of MAYF in Venezuela and, therefore, further documents the public health importance of this disease.
